# Investigation of a bacterial meningitis cluster in a refugee settlement, Obongi District, Uganda, March 2023

**DOI:** 10.11604/pamj.2024.47.11.42377

**Published:** 2024-01-10

**Authors:** Brian Agaba, Rebecca Akunzirwe, Leah Naluwagga Baliruno, Helen Nelly Naiga, Paul Okello, Daniel Kadobera, Lilian Bulage, Richard Migisha, Alex Riolexus Ario

**Affiliations:** 1 Uganda Public Health Fellowship Program, Uganda National Institute of Public Health, Kampala, Uganda

**Keywords:** Bacterial meningitis, cluster, refugee settlement, Uganda

## Abstract

On 6 March 2023, Neisseria meningitidis serogroup C was isolated from a cerebral spinal fluid sample from Obongi District, Uganda. This sample was one of many from patients who were presenting with fever, convulsions, and altered consciousness. We investigated to determine the scope of the meningitis cluster, identify risk factors of contracting meningitis, and inform control measures. We reviewed medical records, conducted active community case finding, and conducted key informant interviews in the affected communities to identify cases and factors associated with contracting meningitis. We analysed case data by person, place, and time. Between 22 December 2022 and 1 May 2023, 25 cases with 2 deaths of bacterial meningitis occurred in Palorinya Refugee Settlement, Obongi District. Of these, 4 were laboratory-confirmed with Neisseria meningitidis serogroup C, 6 were probable cases, and 15 were suspected cases. Most (76%) of case-patients were <18 years old with a median age of 12 years (range 1-66 years). None of the case-patients was vaccinated against Neisseria meningitidis serogroup C. Each case-patient was from a different household and there was no epidemiological link between any of the cases. This meningococcal meningitis cluster caused by Neisseria meningitidis serogroup C occurred among non-vaccinated persons mostly aged <18 years in Palorinya Refugee Settlement. We recommended vaccination of at-risk persons.

## Introduction

Bacterial meningitis is a clinical syndrome characterized by inflammation of the meninges that cover the brain and spinal cord. Up to 95% of patients have at least 2 of the 4 following symptoms: fever, headache, stiff neck, or altered mental status/convulsions [1]. Bacterial meningitis is commonly caused by *Streptococcus pneumoniae, Haemophilus influenzae*, and *Neisseria meningitidis*. Currently, most outbreaks are now caused by *Neisseria meningitidis* [2,3]. With human being the reservoir, bacterial meningitis is transmitted through direct contact from person to person through droplets of respiratory or throat secretions from infected people. The incubation period ranges from 2-10 days with an average of 3-4 days [4]. Case fatality rate ranges from 5-15% with up to 20% of survivors suffering long-term complications such as hearing loss, seizures, limb weakness, difficulties with vision, speech, language, and memory [4].

Uganda lies in the meningitis belt of sub-Saharan Africa and experiences frequent outbreaks of bacterial meningitis. An analysis of surveillance data between 2015 and 2018 showed that the incidence of bacterial meningitis in Uganda was on the increase [5]. In Uganda, the most susceptible regions include West Nile, Bunyoro, Acholi, Lango, Teso, and Karamoja regions [5].

Meningitis outbreak response in the meningitis belt of sub-Saharan Africa encompasses strengthening surveillance for early outbreak investigation, strengthening case management, reactive vaccination of susceptible persons early on in the outbreak, and routine mass vaccination campaigns in areas at greatest risk [6]. As per the Uganda national technical guidelines for Integrated Disease Surveillance and Response (IDSR), bacterial meningitis is one of those diseases that must be reported immediately [7]. These guidelines stipulate two thresholds for public health action with regard to bacterial meningitis. The alert threshold is reached when there are 2 cases per week in an area with less than 30,000 inhabitants or an attack rate of 5 cases per 100,000 inhabitants per week in an area with a population of 30,000 to 100,000 inhabitants. The epidemic threshold is reached when there are 10 suspected cases per 30,000 - 100,000 inhabitants per week or 5 suspected cases in one week in an area with less than 30,000 inhabitants [7]. Despite repeated outbreaks and the availability of national/international guidelines, and efforts to control bacterial meningitis remain suboptimal for many of the countries in the meningitis belt leading to frequent outbreaks [8].

On 6 March 2023, the Uganda Central Public Health Laboratory isolated *Neisseria meningitidis* from a cerebral spinal fluid sample from Obongi District, Uganda. This sample was one of many from patients who were presenting with fever, convulsions, and altered consciousness. We investigated to determine the occurrence, scope, and magnitude of a meningitis outbreak, identify risk factors for contracting meningitis, and inform control/prevention measures.

## Methods

**Figure 1:**
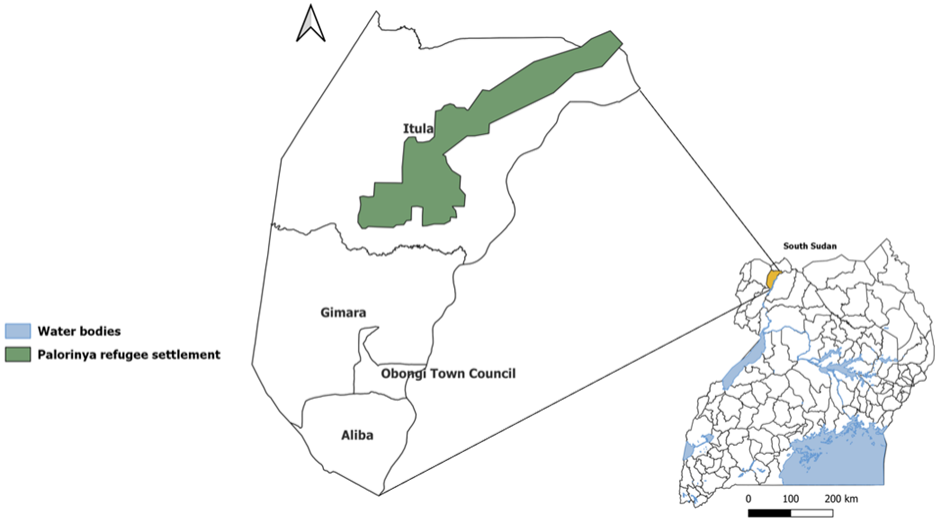
map of Obongi District showing location of Palorinya Refugee Settlement

**Study setting and population:** Obongi District is located in West Nile, one of the regions of Uganda located within the extended meningitis belt of sub-Saharan Africa. It is bordered by Moyo District to the north, Adjumani District to the east, Yumbe District to the west, and Madi Okollo District to the south (Figure 1). The district has a population of 173,325 with 122,000 (70%) being refugees from South Sudan. These refugees have occupied Palorinya Refugee Settlement since 2016. This settlement spans an area of 37.58 Km^2^ and is comprised of 31 villages across two sub-counties of Obongi District.

Palorinya Refugee Settlement is located about 30 kilometres from the South Sudan border. There is frequent movement of people between the settlement and South Sudan. Currently, there are no ongoing vaccination efforts of refugees against meningitis at entry or while in the settlement. Obongi District last carried out a vaccination campaign in 2017 against *Neisseria meningitidis* serogroup A. The district has never vaccinated people against *Neisseria meningitidis* serogroup C.

**Case definition:** we defined a suspected case as a resident of Palorinya Refugee Settlement with sudden onset of fever (>37.5 °C), and any one of neck stiffness, convulsions, altered consciousness, coma or bulging fontanelle in infants from 1 December 2022 to 1 May 2023 [7].

A probable case was any suspected case with macroscopic aspect of cerebrospinal fluid (CSF) turbid, cloudy or purulent; or with a CSF leukocyte count >10 cells/mm^3^ or with bacteria identified by gram stain in CSF; or positive antigen detection (for example, by latex agglutination testing) in CSF. In infants, a probable case was CSF leucocyte count >100 cells/mm^3^; or CSF leucocyte count 10-100 cells/mm3 and either an elevated protein (>100 mg/dl) or decreased glucose (<40 mg/dl) level. A confirmed case was any suspected/probable case that is laboratory confirmed by culturing or identifying (polymerase chain reaction) a bacterial pathogen (*Neisseria meningitidis, Streptococcus pneumoniae, Haemophilus influenzae* type b) in the CSF or blood [7].

**Case finding, data collection, and variables:** we conducted active case search in the community in Palorinya Refugee Settlement and neighboring villages with assistance from community health workers and the district surveillance focal person. We further reviewed the medical records of the health facilities serving the affected villages to identify more cases.

We interviewed the suspected case-patients to identify factors likely associated with contracting the infection. We explored these factors: travel to South Sudan, vaccination against meningitis, and household size. Information on symptoms and signs, date of onset, date of presentation/admission to hospital, duration of illness, drugs, laboratory results, and medical complications was obtained by interviewing the case-patients and reviewing their medical records.

We conducted key informant interviews with the district health officer, Palorinya Refugee Settlement leadership, district surveillance focal person, health facility leaders, and the community health workers of the affected villages to identify possible sources and factors associated with contracting bacterial meningitis.

**Data analysis:** we calculated proportions to describe the distribution of cases by age, sex, and symptoms. We also described case-patients by time of onset of symptoms using an epidemiological curve and calculated attack rates to describe the distribution of cases by place (village). We calculated the time from symptom onset to presentation at the health facility.

**Ethical considerations:** this investigation was in response to a public health emergency and was therefore determined to be non-research. The Ministry of Health gave a directive to investigate this possible outbreak. The authors sought permission to conduct the investigation from the district health authorities of Obongi District, administrators of Palorinya Refugee Settlement, and the health facilities. The authors sought verbal informed consent from the respondents who were at least 18 years old as well as those who were below 18 years of age and emancipated. The authors also sought assent from children below 18 years of age who were not emancipated and informed verbal consent from their parents or guardians.

This activity was reviewed by Centres for Disease Control and Prevention (CDC) and was conducted consistent with applicable federal law and CDC policy (§§See e.g., 45 C.F.R. part 46, 21 C.F.R. part 56; 42 U.S.C. §241(d); 5 U.S.C. §552a; 44 U.S.C. §3501 et seq).

## Results

**Figure 2:**
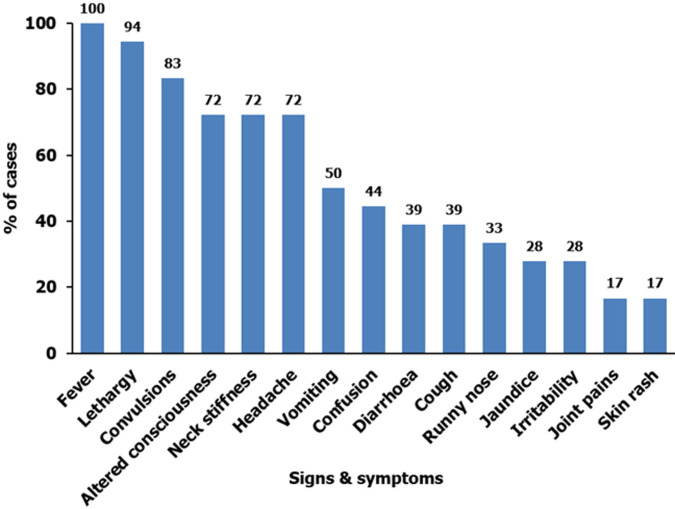
distribution of case-patients by symptoms during the bacterial meningitis cluster in Palorinya Refugee Settlement, Obongi District, Uganda, December 2022 - May 2023

**Descriptive epidemiology:** we line listed a total of 25 cases of bacterial meningitis. Of these, 4 were confirmed cases, 6 were probable cases, and 15 were suspected cases. The case fatality rate was 8% (2/25). The median age of the case-patients was 12 years (range 1-66). Most (76%) of the case-patients were <18 years. Case-patients presented with fever (100%), lethargy (94%), convulsions (83%), headache (72%), neck stiffness (72%), altered consciousness (72%), vomiting (50%), confusion (44%), diarrhea (39%), cough (39%), and runny nose (33%) (Figure 2). In this cluster, 2/25 case-patients reported complications related to meningitis (hearing loss (1/25) and limb weakness (1/25)).

The overall attack rate (AR) for bacterial meningitis in Palorinya Refugee Settlement was 21/100,000 population. Persons aged 12-17 years were the most affected (AR 43/100,000) followed by 1-4 years (AR 33/100,000), >59 years (AR 20/100,000), 5-11 years (AR 15/100,000), 18-59 years (AR 10/100,000). Males (AR 30/100,000) were more affected than females (AR 12/100,000). All weeks in which meningitis cases were reported had attack rates below the epidemic threshold of 10 cases per 100,000.

**Figure 3:**
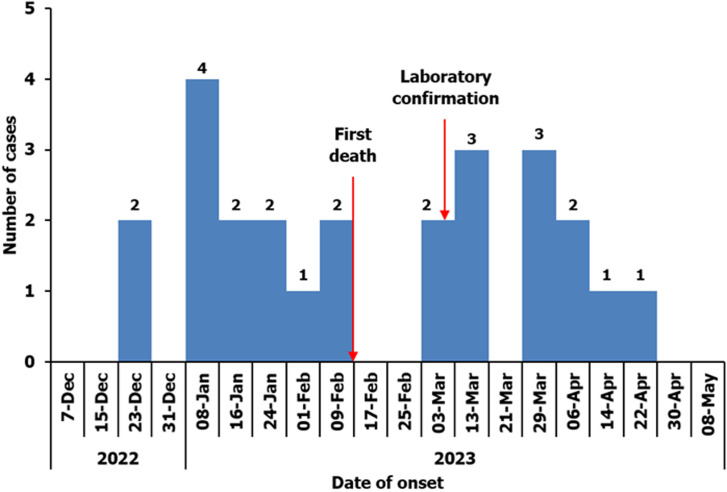
distribution of bacterial meningitis cases by date of symptom onset in Palorinya Refugee Settlement, Obongi District, Uganda, December 2022 - May 2023

The median household size was 7 (range 1 to 10) people with 3 (range 1 to 6) people sharing a bedroom. None of the case-patients had travelled out of the refugee settlement in the six months prior to illness. All case-patients received health care from government health facilities. The majority (83%) of case-patients sought medical care within 24 hours of symptom onset. All case-patients were treated with ceftriaxone antibiotic. This cluster of bacterial meningitis started on 22 December 2022 and ended on 1 May 2023. The first case of meningitis occurred on 22 December 2022 and laboratory confirmation of meningitis was on 6 March 2023 (Figure 3).

As of May 1^st^, 2023, nine villages had reported cases of bacterial meningitis. Seven of the villages were in Palorinya Refugee Settlement while two of the villages were neighboring the settlement. The affected villages were scattered all over the refugee settlement. Some affected villages were more than 20 kilometers apart. Luakoke (AR=79/100,000), Belameling (AR=65/100,000), and Keguru (AR=64/100,000) were the most affected villages (Table 1).

**Table 1:** attack rate by village during the bacterial meningitis cluster in Palorinya Refugee Settlement, Obongi District, Uganda, December 2022 - May 2023

Village	Cases	Population	Attack rate/100,000
Luakoke	2	2,539	79
Belameling	5	7,737	65
Keguru	6	9,378	64
Luru	3	7,649	39
Idiwa	4	12,354	32
Dongo	1	4,720	21
Pasu	1	9,337	11
Kali	2	NP	-
Umbechi	1	NP	-

NP: population data not available

**Factors likely associated with contracting meningitis infection:** authorities reported frequent to and from movement of persons from South Sudan and suspected that the cases could be associated to the movement. Although authorities suspected that this cluster could have been imported from South Sudan, there was no evidence to point to that, they had not contacted their counterparts in South Sudan to find out whether there was an ongoing meningitis outbreak.

None of the case-patients was vaccinated against *Neisseria meningitidis* serogroup C. Only 2/25 case-patients reported a history of vaccination. This was in 2017 with a vaccine against *Neisseria meningitidis* serogroup A.

## Discussion

This cluster of meningitis caused by *Neisseria meningitidis* serogroup C was confined to Palorinya Refugee Settlement and neighbouring villages. Although the cluster started on 22 December 2022, the Ministry of Health was alerted ten weeks later on 6 March 2023. We line listed 25 cases with a case fatality rate of 8% and a complication rate of 8%. The cases were sporadically distributed across an area of 20 Km^2^ with no epidemiological link between them. No household reported more than one case. Case-patients had good health-seeking behaviour and were all appropriately treated with effective antibiotics. There was frequent in and out movement of persons between South Sudan and the Palorinya Refugee Settlement. There was no vaccination campaign against bacterial meningitis in the district in the last 5 years.

Although the first case of meningitis presented to the health facility on 22 December 2022, the Ministry of Health was only alerted 10 weeks later. According to the Uganda national technical guidelines on Integrated Disease Surveillance and Response [7], bacterial meningitis is one of the diseases requiring immediate reporting. Surveillance forms the backbone of bacterial meningitis control. Official declaration of a bacterial meningitis outbreak, resource mobilisation, effective case management, and reactive mass vaccination campaigns all depend on an efficient surveillance system [9,10]. Our investigation revealed a gap in surveillance that needs to be addressed to better protect the at-risk communities against bacterial meningitis.

This cluster of meningitis was caused by *Neisseria meningitidis* serogroup C. Studies have shown that serogroup C is becoming a major cause of meningococcal meningitis in the meningitis belt of sub-Saharan Africa [11,12]. This is because there have been minimal vaccination campaigns against serogroup C leaving at-risk populations susceptible to this serogroup. As of 1 May 2023, there was no documented vaccination campaign against serogroup C in Uganda. In contrast, other causes of bacterial meningitis have been targeted through routine immunisation of children with *Haemophilus influenzae* and pneumococcal vaccines. In 2017, there was a vaccination against *Neisseria meningitidis* serogroup A in Obongi and 38 other districts in Uganda. This could explain why the other causative organisms for bacterial meningitis are declining while serogroup C is increasing. Mass vaccination campaigns have shown efficacy in the meningitis belt of Africa [4,13].

The case fatality rate (8%) and the complication rate (8%) of bacterial meningitis in this outbreak were low. Studies show that 5-15% of bacterial meningitis cases die while 20% of survivors suffer long-term complications such as hearing loss, seizures, limb weakness, difficulties with vision, speech, language, and memory [4,14]. In this outbreak, 83% of case-patients received medical care within 24 hours of symptom onset and were all treated appropriately with an effective antibiotic. This could explain the low case fatality and complication rates seen in this outbreak. Without treatment, case fatality rates for bacterial meningitis may increase up to 50% [15].

Our investigation failed to establish an epidemiological link between any of the case-patients. None of the affected households had more than one case. Studies show that most cases of bacterial meningitis are spread from asymptomatic carriers [16]. Literature on the epidemiology of bacterial meningitis shows that 10% to 20% of the population carries *Neisseria meningitidis* in their throat at any given time [15]. However, less than 1% of persons colonised with *Neisseria meningitidis* progress to invasive disease [17-19]. It is likely that these case-patients acquired the infection from asymptomatic carriers.

**Public health actions:** the results from this investigation were shared with the Ministry of Health National Task Force, Palorinya Refuge Settlement leadership, and Obongi District health authorities. As a result, at the national level, a technical working group was setup to monitor the cluster/outbreak and establish a working relationship with South Sudan health authorities to share information. At district level, surveillance activities were continued until 2 weeks after the last case. We supported the district to make daily situation reports of the outbreak.

**Study limitations:** this investigation was started 10 weeks after the first case of bacterial meningitis was presented to the hospital. As a result, our findings could be affected by recall bias. Case-patients were reluctant to discuss travel in and outside the refugee settlement and felt this could jeopardize their refugee status. This could have led to social desirability bias.

## Conclusion

This cluster of meningococcal meningitis caused by *Neisseria meningitidis* serogroup C did not reach the epidemic thresh hold stipulated in the IDSR guidelines. None of the affected persons was vaccinated against *Neisseria meningitidis* serogroup C. We recommend strengthening meningitis surveillance through cross-border collaboration between Uganda and South Sudan, training of health workers in case detection and reporting. Mass vaccination of at-risk persons with vaccines targeting common *Neisseria meningitidis* serogroups (A, B, C, W, Y) could reduce the magnitude, case fatality, and complication rates of outbreaks.

### What is known about this topic

- *Cases of bacterial meningitis are common in areas that lie in the meningitis belt of Africa*.

### What this study adds

- *Neisseria meningitidis serogroup C is an increasing cause of bacterial meningitis in the meningitis belt of Africa;*
- *Cases may occur with no epidemiological link suggesting that spread is mostly from asymptomatic carriers*.
